# Adsorption of Safranin O Dye by Alginate/Pomegranate Peels Beads: Kinetic, Isotherm and Thermodynamic Studies

**DOI:** 10.3390/gels9110916

**Published:** 2023-11-18

**Authors:** Amina Abbaz, Sihem Arris, Gianluca Viscusi, Asma Ayat, Halima Aissaoui, Yasser Boumezough

**Affiliations:** 1 Environmental Process Engineering Laboratory (LIPE), Faculty of Process Engineering, Salah Boubnider University Constantine 3, Constantine 25000, Algeria; asma.ayat@univ-constantine3.dz (A.A.); halima.aissaoui@univ-constantine3.dz (H.A.); yasser.boumezough@univ-constantine3.dz (Y.B.); 2 Department of Industrial Engineering, University of Salerno, Via Giovanni Paolo II, 132, 84084 Fisciano, Italy; gviscusi@unisa.it

**Keywords:** alginate, pomegranate, cationic dye, adsorption, adsorbent, isotherm

## Abstract

Water pollution is regarded as a dangerous problem that needs to be resolved right away. This is largely due to the positive correlation between the increase in global population and waste production, especially food waste. Hydrogel beads based on sodium alginate (Alg) and pomegranate fruit peels (PP) were developed for the adsorption of Safranin O dye (SO) in aqueous solutions. The obtained Alg−PP beads were widely characterized. The effects of the contact time (0–180 min), initial concentration (10–300 mg/L), initial pH (2–10), adsorbent dosage (1–40 g/L) and the temperature (293–333 K) were investigated through batch tests. The data proved that the adsorption kinetics of SO reached equilibrium within 30 min and up to 180 min. The dye adsorption is concentration dependent while a slight effect of pH was observed. The adsorption data of SO onto synthesized beads follow the pseudo second-order model. The experimental data fitted very well to Langmuir model with correlation factor of 0.92 which demonstrated the favourable nature of adsorption. The maximum adsorption capacity of Alg−PP could reach 30.769 mg/g at 293 K. Calculation of Gibbs free energy and enthalpy indicated that adsorption of SO onto Alg−PP is spontaneous (negative ΔG) and endothermic (ΔH = 9.30 kJ/mol). Analysis of diffusion and mass transport phenomena were presented. The removal efficiency was found to be 88% at the first cycle and decreased to 71% at the end of the seventh cycle. The reported results revealed that the Alg−PP beads could be used as a novel natural adsorbent for the removal of high concentrated solutions of Safranin O which is a cationic dye from liquid affluents and as future perspective, it can be used to remove various pollutants from wastewater.

## 1. Introduction

Many different types of pollution affect water resources, harming aquatic organisms. Pollutants can be produced naturally, but a large portion is due to human activities like industrial processes [1]. Many companies use dyes during the production processes [2]. Even at low concentrations, the discharge of effluents containing dyes is highly visible and has undesirable effects. The primary effect is the decrease in sunlight penetration throughout the water body, which influences photosynthetic activity and, as a result, the rate of dissolved oxygen. The synthetic dye consumption in textile manufacturing is approximately 10,000 tons per year, and this amount will produce approximately 100 tons of contaminants in wastewaters [3]. The presence of a trace amount of dyes in wastewater (˂1 mg/L) is unpleasantly abhorrent and inconvenient [4]. One of the most common one is the cationic Safranin-O dye. Safranin O (SO, commonly known as basic red 2) is a dye used in the histology and cytology fields of the pharmaceutical and biochemical industries. It is frequently employed for detecting cartilage, mucin, and mast cell granules as well as staining gram-negative bacteria [5]. Furthermore, it is used less frequently in food products, as well as in the coloring and treatment of tannins, cotton, fibers, wool, paper, and leather [6]. In addition, SO may cause adverse health effects such as eye irritation, dermatitis, and respiratory allergies [7]. Given these consequences, appropriate wastewater treatment is required. Thus, the effective removal of this dye has become important and, in that context, its adsorption/bio adsorption/photodegradation on several heterogeneous surfaces has been investigated to minimize the exposure of this dye to health and the environment. In this regard, there is a great incentive to develop novel and cheap technologies [6]. Although many techniques, including membrane filtration, are suggested for dye removal [8] (such as ion exchange [9], coagulation-flocculation [10], precipitation [11], and electrocoagulation [12]), adsorption is an interesting alternative [13]. Since the high cost of some typical adsorbents, adsorption techniques using unconventional low-cost materials from renewable and cheap biomass or industrial wastes have been investigated to overcome such disadvantages and improve economic viability [14]. Agricultural wastes have the benefits of being accessible, cost-effective, and environmentally friendly in addition to being biodegradable, non-toxic, and cheap [15,16,17,18]. Many natural and low-cost adsorbents are now being used successfully in the removal of pollutants from aqueous solutions such as peanut hull [19], rice straw [20], date stone [21], forest residue [22], natural fibers [23], pulp and paper [24] and cotton stalks [25]. So far, different agro waste resources have been exploited to remove safranin O such as pineapple peels [26], orange peels [27], mandarin peels [28], coconut coir [29], blueberry seeds [30], rice straw [31], tea waste powder [32], olive leaves powder [33]. Pomegranate peel waste has been successfully used as an efficient, cost-effective for the removal of pollutants from wastewater [34,35,36]. Pomegranate, belonging to the Punica L. genus, *Punicaceae* family, is widely cultivated worldwide. It is a byproduct of the pomegranate juice industry, wine industries, and tanneries [37]. The production and consumption of pomegranates keep increasing. The total worldwide pomegranate production is estimated to be around one 1.5 million tons [38]. However, when processed, a large amount of waste is generated, in which peels take up about 26–30% of the total weight, causing a problem of waste disposal and management [39] which is a major environmental concern for producer countries (such as India, Iran, Turkey, the United States, China, Spain, and Morocco) [40,41]. In Algeria, the pomegranate cultivation has seen an increase in recent years with an area increasing from 8386 hectares for a production of 58,601 tons in 2006 to an area of 11,510 ha for a production of 84,867.6 tons in 2017. (Statistica 2023). To address this issue, various revalorization methods, such as the production of valuable compounds for essential oils [42,43], food additives [44], and medicinal products [45], as well as energy and value-added products such as bioethanol and biogas, have been introduced. Apart from active compounds such as polyphenols, dietary fiber, vitamins, minerals the organic waste could be used to produce novel bio-based adsorbent for water remediation. As a lignocellulosic material, it contains large amounts of functional groups (e.g., –OH, –COH) which give them an ion exchange capacity and general adsorptive characteristics [34]. Due to that, PP is being used more and more as a bio-adsorbent for heavy metals, dyes, and other contaminants [46,47]. So far, few studies have investigated the potential capacity of modified pomegranate peel for wastewater purification, especially untreated peels. Despite the effectiveness of these low-cost adsorbents, there are some issues related to their implementation, such as the difficulty of their regeneration and the separation of treated water. The use of biopolymers as carrier to encapsulate these materials could be considered a solution to this issue [48]. Developing effective, economical, and friendly strategies to tackle environmental pollution has been a hotspot in recent years, among which adsorption of chemical pollutants from water has been considered of utmost importance. Hydrogel-based adsorbents with three-dimensional porous structures and versatile functional groups have become the first choice, whose adsorption involves several mechanisms. Beads adsorbents are easy to separate, recycle, and have low secondary pollution, which makes them good adsorbent in the field of wastewater treatment [49]. Many based adsorbents, particularly composite hydrogels, were developed to meet the requirements of sustainable development for the adsorption of dyes in wastewater such as activated carbon prepared from waste peanut shell [50], graphene oxide [51], flax seed ash [52], activated lemon peels [53], bentonite [54], activated-organo bentonite [55], kelp biochar [56], calcium alginate bead modified with polyethyleneimine [57], nano-silver/diatomite [58]. Therefore, the creation of adsorbent composite beads can be used in water treatment processes, particularly continuous ones as opposed to discontinuous ones [59,60,61].

The most popular polymer for this purpose is alginate, which is an anionic and hydrophilic polymer found mostly in brown algae [62]. Alginate is a natural polysaccharide extracted from brown seaweed with several properties that make it suitable for the adsorption process [63]; one of which is the gelation property, which allows for combining various substances through an egg-box structure [64]. Alginate has many advantages such as availability, low cost, non-toxicity, biocompatibility, and biodegradability, and is also an efficient biosorbent due to the presence of carboxylate functions along its chains. It has been used in a variety of industries over the last decade, including pharmaceutical (conventional use) [65], food [66], and increasingly as coagulants and sorbents for dyes removal [67,68]. Many studies in recent years have shown that hydrogels based on SA have good adsorption capacities for pollutants such as metal ions, antibiotics, and organic pollutants in aqueous media and are effective for wastewater treatment [69,70]. Furthermore, numerous studies have successfully incorporated functional materials such as activated carbon to improve its sorption capacity [71] or maghemite nanoparticles [72] to improve its separation ability. According to the findings of the previous studies, composite alginate beads are promising adsorbents for the remediation of aqueous solutions contaminated with dyes. Furthermore, reusability tests have shown that the adsorption capacity persists after four to ten consecutive adsorption-desorption cycles [52,53,57,58]. However, the low physical strength and thermostability of sodium alginate-based materials have significantly restricted their use in industrial applications.

Based on the previously reported statements, the beads of multi-functional core-shell pomegranate peel amended was performed in adsorption of phenol from wastewater [73]. This is the first investigation focused on the adsorption of dye which is safranin O with pomegranate peels that is encapsulated in alginate beads. Conventional adsorbents are generally difficult to regenerate so they are often used only once. The encapsulation of pomegranate peel powder, which have already proven their effectiveness, in alginate beads makes them easy to regenerate and therefore exploit them in several adsorption cycles. The adsorbent was widely characterized while the effects of different parameters on the removal of SO process were investigated. Finally, the adsorption kinetics, isotherms, thermodynamics, and regeneration ability of the hydrogel beads were studied. The outcomes prove the feasibility of using the designed systems for removing high concentrated SO from wastewaters. As future perspective, the produced beads systems could be potentially used to remove other kinds of pollutants from wastewater.

## 2. Results and Discussion

### 2.1. Characterization

#### 2.1.1. Morphology, Size and Water Content of Beads

**Figure 1:**
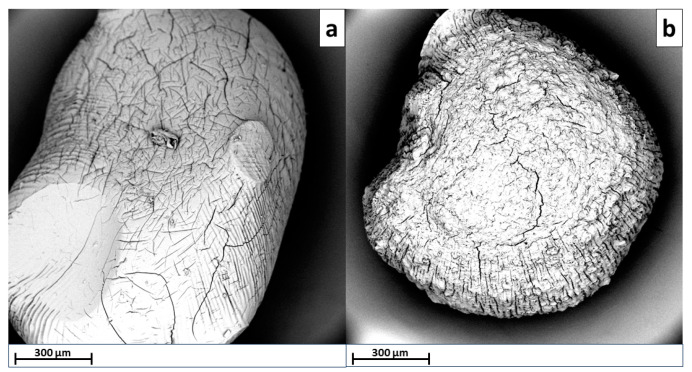
SEM micrographs of Alg (**a**) and Alg−PP (**b**).

Figure 1 reports the SEM micrographs of Alg (a) and Alg−PP (b).

The SEM photographs of the beads show that the Alg beads possess a smooth surface with compact structure. Besides, after loading PP, the beads appear to be rougher with cracks and pores due to the presence of lignocellulosic material. Besides, corrugations and wrinkles are evident. The PP loaded beads showed a high porosity, coarse and irregular structure with cavities and superficial fractures which could affect the uptake of dye molecule.

**Figure 2:**
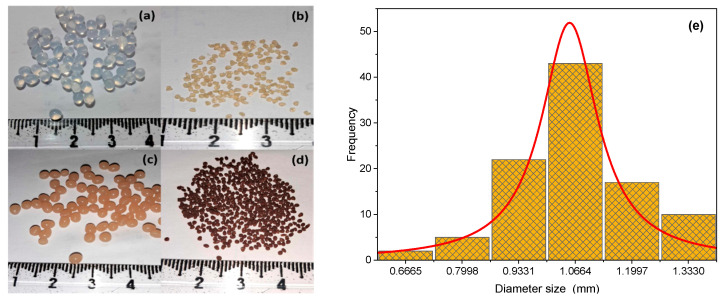
Photographs of wet pure Alg beads (**a**), dried pure Alg beads (**b**), wet Alg−PP beads (**c**), dried Alg−PP (**d**), size distribution (diameter) of the dried Alg−PP beads (**e**).

Pictures of beads are reported in Figure 2. The Alg−PP beads appeared brown, roughly spherical-shaped with a millimetric size (Figure 2a), while the mean diameter of the dried beads was 1 ± 0.4 mm (Figure 2b). The diameter distribution of the Alg−PP beads is shown in Figure 2e. Finally, the water content of beads was found to be 95.13 ± 2.4%.

#### 2.1.2. Swelling Ratio

The water swelling ratio of Alg beads was found to be 16.6% while, for Alg−PP a value of 53.9% was recorded. Compared to pure Alg, this increase in water swelling ratio of Alg−PP gel beads can be associated to their higher surface area. Besides, the higher swelling ratio value for Alg−PP sample is related to its higher porosity and the more hydrophilic structure of PP powder [27,41]. Therefore, the swelling ratio studies also suggest that the Alg−PP beads is more suitable to adsorb dye molecules.

#### 2.1.3. FTIR Analysis

**Figure 3:**
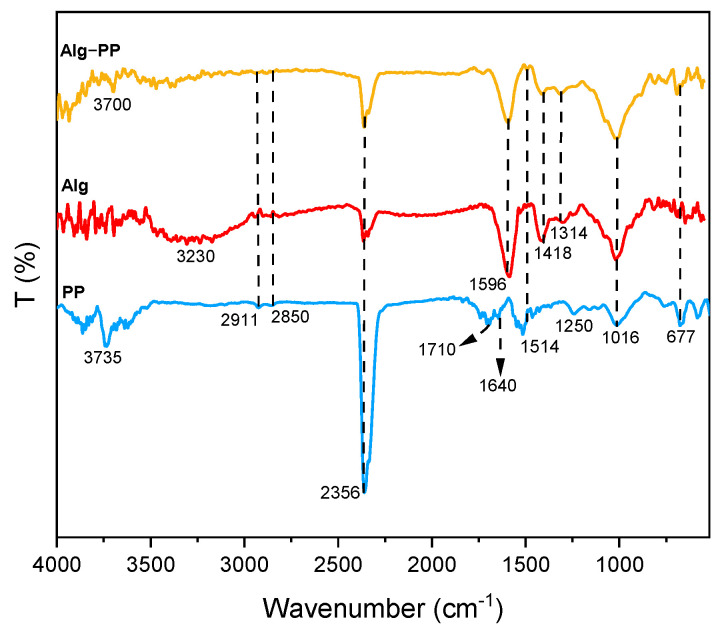
FTIR spectra of PP, Alg and Alg−PP.

The analysis of FTIR gives more insight into the presence of the functional groups on the surface of the PP and the hydrogel beads (Figure 3).

For Alg spectrum, broad peaks centered at 3230 cm^−1^ indicated the presence of hydroxyl (O−H) stretching vibrations [74]. Besides, PP and Alg−PP didn’t show any broad peaks apart from medium peaks at higher wavelengths associated to the presence of O−H stretching functional groups. Peak located at 2911 cm^−1^ and 2850 cm^−1^ are related to C−H stretching of CH_2_ in the PP, Alg and Alg−PP. The observed peak at 1710 cm^−1^ in PP spectra corresponds to the C=O stretching of the aldehydes, ketones, and carbonyl groups. The peak around 1596 cm^−1^ and 1418 cm^−1^ can be assigned to the symmetric and antisymmetric stretching vibrations of COO^−^ functional groups [75], the first one clearly overlapping δ_OH_ vibration modes of adsorbed molecules of water. The peak at 1514 cm^−1^ in PP and Alg−PP represents the cellulosic compounds present in the biomaterial [76]. The absorption band at 1314 cm^−1^ were assigned to the CH stretching vibrations. Additionally, the band at 1016 cm^−1^ is associated with the C−O stretching vibration of lignin and hemicelluloses components [77].

#### 2.1.4. TGA Analysis

**Figure 4:**
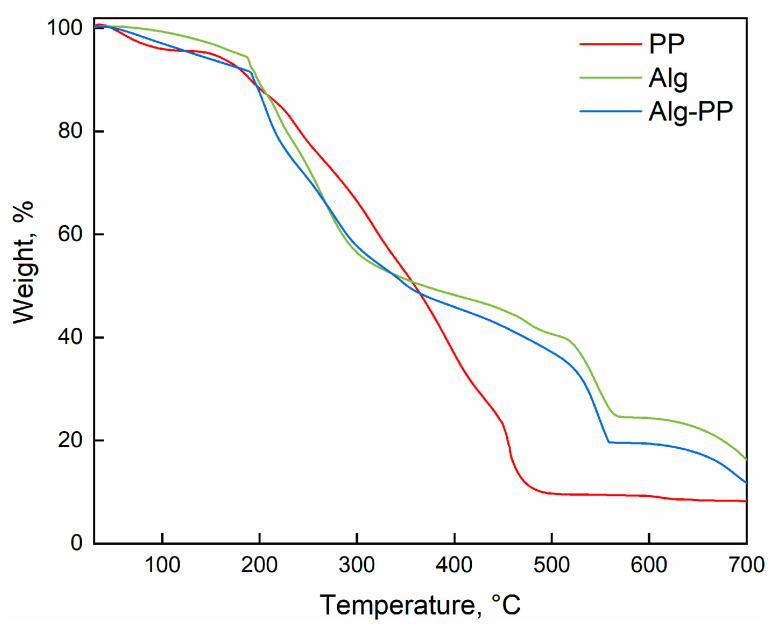
TGA analysis of PP, Alg and Alg−PP.

Figure 4 reports the TGA profiles of PP, Alg and Alg−PP.

The thermal decomposition profiles show different thermal steps. At low T, evaporation of physical and bonded water is supposed to occur. From 200 to 300 °C, the breaking of the alginate backbone and the loss of OH groups due to the dehydration could happen [78,79]. Above 300 °C, sodium alginate tends to decarboxylate releasing carbon dioxide and other volatile gases [17]. The TGA profile of PP shows the typical behavior or cellulosic material. It is supposed to be a lignocellulosic biomass comprising cellulose, hemi-cellulose as well as lignin [80]. The first step is attributed to water evaporation. The next degradation steps could be explained by organic material decomposition [77]. The last step concerns the carbonization indicating that the decomposition of organic materials was completed. Finally, a lower amount of residue for Alg−PP was obtained compared to pure Alg beads.

### 2.2. Adsorption Tests

#### 2.2.1. Effect of Contact Time and Initial Dye Concentration

**Figure 5:**
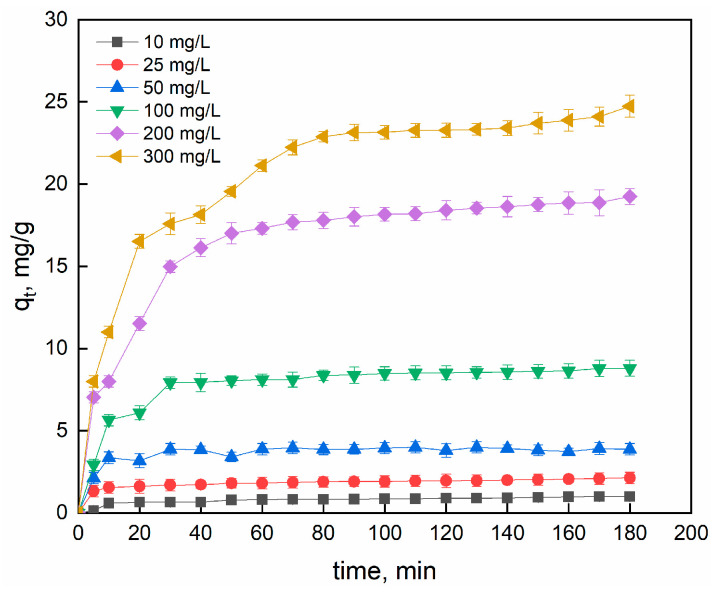
Effect of initial concentration on adsorption capacity.

Figure 5 shows the effect of contact time and initial dye concentration on adsorption (r = 10 g/L).

The adsorption trend was essentially separated into three stages: initial rapid adsorption, slowing of adsorption rate, and eventually achievement of equilibrium state. The adsorbent surface contains active sites, so the SO molecules are accumulated on the monolayer of the adsorbent surface. Pores and active sites were saturated as the adsorption time increased. This is evident in the second step, and the adsorption capacity slowly increases before levelling off. The SO molecules can then penetrate the pores in the final region, before reaching a steady situation due to the repulsive forces between the free SO molecules in bulk solution and the adsorbed molecules [81]. The results indicated that the maximum uptake capacities were 0.99, 2.13, 3.99, 8.79, 18.87 and 24.73 mg/g for the 10, 25, 50, 100, 200 and 300 mg/L Safranin O solutions, respectively. The time required to reach plateau regime was reached after 20 min for C_0_ = 10, 25, 50 and 100 mg/L SO and after 40 min for higher concentrations. The dye adsorption is concentration-dependent due to the higher initial concentration which contributed to increase the driving force overcoming the mass transfer resistance [82]. Where the initial concentration C_0_ = 50 mg/L the adsorption efficiency was 88% with a residual concentration in the aqueous solution of 6 mg/L. Since the final concentration is still high, we suggest conducting a second adsorption cycle (successive adsorption) to reduce it to standard.

#### 2.2.2. Effect of Initial pH

**Figure 6:**
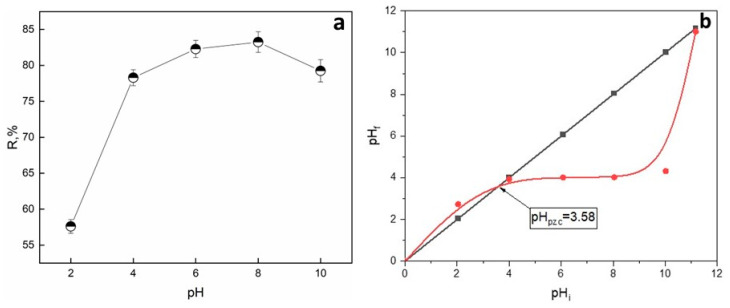
(**a**) Effect of the initial pH on the removal of SO; (**b**) the point of zero charge of Alg−PP beads.

The pH plays a crucial role since it can affect the surface charge of the adsorbent as well as the adsorbate properties. The pH effect was studied at the following conditions: dosage of 500 mg and an initial dye concentration of 50 mg/L (Figure 6a).

pH seems to have a slight effect on the adsorption of SO onto Alg−PP. The percent of SO removal increased from 68% to 85% when the pH changed from 2 to 10. It is observed that maximum dye removal occurs at pH 10. Moreover, even at the lowest pH, the R% is roughly 70% since the presence of active sites. To evaluate the effect of pH on the SO adsorption, the point of zero charge (pH_pzc_) of the adsorbent must be investigated (Figure 6b). The pH_pzc_ value for the Alg−PP indicates that the surface of the beads is acidic (3.58). At pH > pH_pzc_ = 3.58 the adsorption of cationic dyes is favoured due to the presence of functional groups such as OH and the formation of electrostatic forces. The lowest SO removal percent is found in the highly acidic mediums (pH < pH_pzc_), due to the electrostatic repulsion between the H^+^ and the cationic SO. In acidic conditions, an amino group (−NH_2_) of safranin-O dye is protonated (pK_a_ = 11), inhibiting the hydrogen bonds formation between SO dye and Alg−PP [83]. At higher pHs, the deprotonated form of safranin-O dye diminishes the possibility of creating a binding between dye molecules and adsorbents [84]. Due to that behaviour, no higher pHs were investigated since the percentage of removal is supposed to decrease.

#### 2.2.3. Effect of Adsorbent Dosage

**Figure 7:**
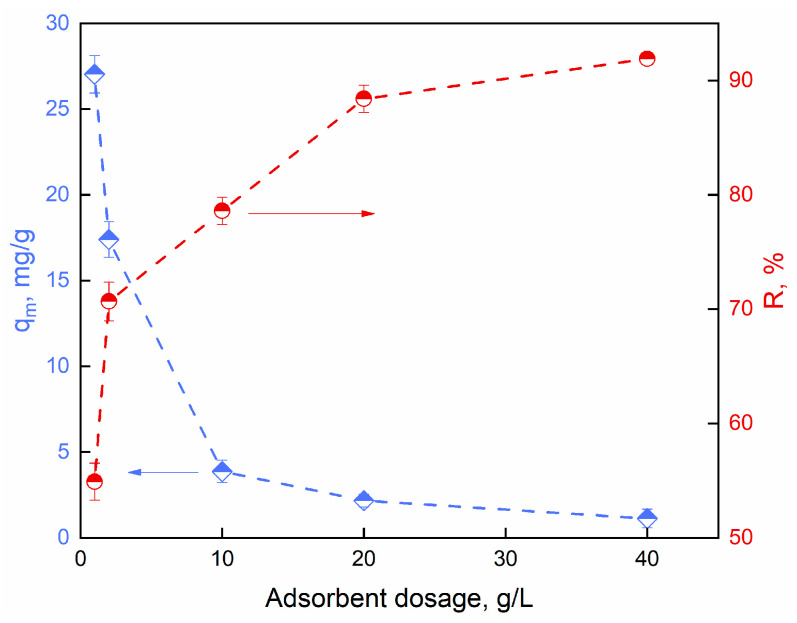
Effect of adsorbent amount on the capacity and the removal of SO adsorption.

Different dosages of Alg-PP beads were put in contact with a constant initial dye concentration of 50 mg/L to study the effect of adsorbent dosage. The % removal and adsorption capacity of dye after a contact time of 3 h are shown in Figure 7. It appeared that the % uptake increased from 58 to 95% when the adsorbent dose was increased from 1 to 40 g/L, respectively. The % uptake reached a maximum value at higher adsorbent dosage. As the adsorbent dosage increases, higher number of adsorption sites are available for the adsorption, leading to improvement of dye removal. Also, with increasing adsorbent load, the quantity of dye adsorbed per weight of the adsorbent gets reduced, thus causing a decrease in q_e_ value with increasing Alg−PP beads loading. Beyond 10 g/L, the R% of safranin O dye may be reduced due to overcrowding of dye molecules since it is prohibited an appropriate binding between the dye groups and adsorbent [83].

### 2.3. Adsorption Kinetics

The adsorption kinetics of SO onto the hydrogel beads were analyzed with pseudo-first-order model (Equation (1)) [85], pseudo-second-order model (Equation (2)) [86], intraparticle diffusion model (Equation (3)) [87] and Elovich model (Equation (4)) [88]:

(1) $$\ln\left(q_{e}-q_{t}\right)=lnq_{e}-k_{1}t$$

(2) $$\frac{t}{q_{t}}=\frac{1}{k_{2}q_{e}^{2}}+\frac{1}{q_{e}}t$$

(3) $$q_{t}=k_{i}t^{1/2}+C$$

(4) $$q_{t}=\frac{1}{\beta }\ln\left(\alpha \beta \right)+\frac{1}{\beta }lnt$$

where $q_{t}$ (mg/g) is the adsorption capacity at time t (min), $q_{e}$(mg/g) is the equilibrium adsorbent capacity, $k_{1}$ (min^−1^), $k_{2}$ (g/mg min) and k_i_ (mg/gmin^1/2^) are the rate constants of the pseudo-first-order, pseudo-second-order and intra-particle diffusion kinetic models, respectively; C (mg/g) is a constant that represents the boundary layer thickness, α is the initial adsorption rate in (mg/g min) and β (g/mg) is the desorption constant correlated with the degree of the chemisorption surface cover and activation energy. 1/β value shows the number of sites available for adsorption while $\frac{1}{\beta }\ln\left(\alpha \beta \right)$ indicates the adsorption quantity.

Finally, the diffusion coefficient of intraparticle diffusion $D_{i}$ for the adsorption of SO onto PP can be calculated using the Equation (5) [89]:

(5) $$D_{i}=\frac{0.03*r^{2}}{t_{1/2}}$$

where $D_{i}$ is the intraparticle diffusion coefficient (cm^2^ s^−1^), $t_{1/2}$ is the time required to complete half the adsorption (s) and r is the beads radius (cm). If the values of $D_{i}$ are in the range of 10^−5^ to 10^−13^ cm^2^/s then intraparticle diffusion is involved as the rate-limiting step, especially for chemisorption systems [89].

**Figure 8:**
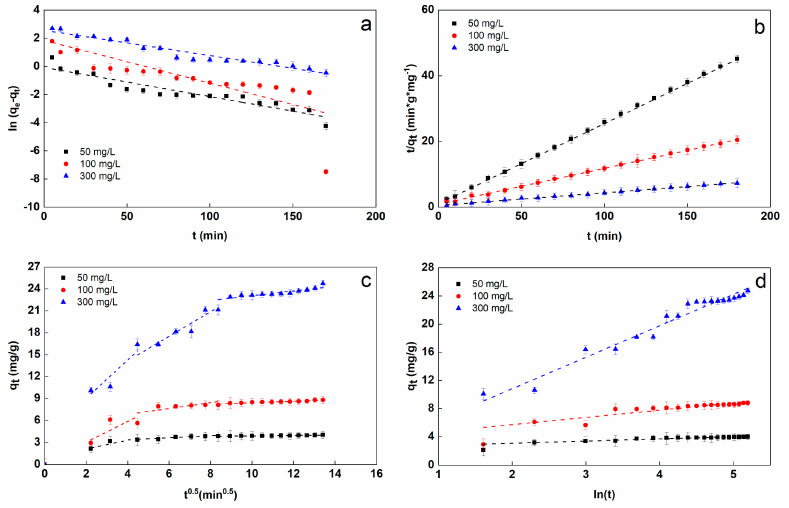
Fitting of pseudo-first-order (**a**), pseudo-second-order (**b**), intraparticle diffusion (**c**) and Elovich (**d**).

Fitting of kinetics models and the derived kinetic parameters for the Alg−PP beads at different initial concentrations are reported in Figure 8 and Table 1. The adsorption of SO dye followed the pseudo-second-order model with best value of R^2^ ≥ 0.9999 and smaller SSE and RMSE, while the theoretical q_e,cal_ values were in accordance with the experimental data. This suggests that chemisorption occurs involving the different interactions between the dye and adsorbent functional groups.

As seen in Figure 8c, the plots of qt against t^1/2^ give three straight-lines with different slopes and intercepts. Based on the k_i_ values, the rate constants related to the external surface adsorption (k_i1_) was the highest one. This also shows that the diffusion resistance of the boundary layer was lower than the diffusion resistance of the pore diffusion. Therefore, the adsorption kinetics may be governed by external diffusion and intra-particle diffusion at the same time [31]. The value of the pore diffusion coefficient $D_{i}$ presented in Table 1 is in the range of 10^−6^ to 10^−8^ cm^2^ s^−1^ indicating that intraparticle diffusion was involved in the adsorption process controlled by chemisorption.

### 2.4. Adsorption Isotherm

**Figure 9:**
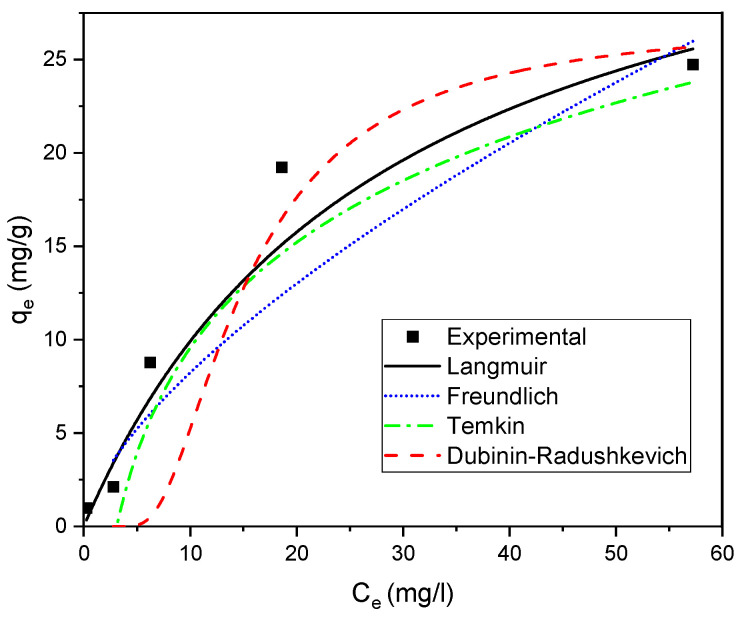
Nonlinear curves of adsorption isotherms.

The adsorption isotherm models allow to analyze the interactions between the adsorbent and the adsorbate. Langmuir [90], Freundlich [91], Temkin [92] and Dubinin-Radushkevich [93] isotherm models are used to describe the adsorption data of SO on Alg-PP. the nonlinear curves are showed in Figure 9.

The Langmuir isotherm model assumes uniform adsorption energies on the adsorbent surface. It is based on the assumption of monolayer adsorption on a completely homogeneous surface with a finite number of identical sites and negligible interaction between adsorbed molecules [90].

The Langmuir isotherm model is reported hereinafter (Equation (6)):

(6) $$q_{e}=\frac{q_{m}bC_{e}}{1+bC_{e}}$$

Equation can be linearized into the following form (Equation (7)):

(7) $$\frac{1}{q_{e}}=\frac{1}{q_{m}}+\frac{1}{bq_{m}}\cdot \frac{1}{C_{e}}$$

where $C_{e}$(mg L^−1^) is the dye concentration at equilibrium, $q_{e}$ (mg g^−1^) is the equilibrium adsorption capacity, $q_{m}$(mg g^−1^) and b (L mg^−1^) are the Langmuir constants.

Freundlich isotherm model (Equation (8)):

(8) $$q_{e}=K_{f}C_{e}^{\frac{1}{n}}$$

The Freundlich model is an empirical equation that is based on the adsorption of heterogeneous surface or surface supporting sites with different affinities. The stronger binding sites are assumed to be occupied first, and the binding strength decreases with increasing site occupancy [91].

Equation (8) can be linearized (Equation (9)):

(9) $$\log q_{e}=\log K_{f}+\frac{1}{n}\log C_{e}$$

where $C_{e}$ and $q_{e}$ are explained as above, $K_{f}$((mg g^−1^)*(L mg^−1^)1/n) and n are the isotherm constants.

The Temkin isotherm assumes that when the layer is covered, the heat of adsorption of all molecules in the phase decreases linearly and that the adsorption has a maximum energy distribution of uniform bond [92].

Temkin isotherm model (Equation (10)):

(10) $$q_{e}=B\ln a_{T}+B\ln C_{e}$$

where *B* = *RT*/*b_T_* and $b_{T}$ the Temkin constant related to sorption heat, $a_{T}$ is the binding equilibrium constant, R (8.314 J mol^−1^ K^−1^) is a standard gas constant and T(K) is the absolute temperature of the solution.

The Dubinin-Radushkevich isotherm was developed as an empirical model for the adsorption of subcritical vapours onto micropore solids via a pore filling mechanism. It is used to differentiate between physical and chemical adsorption when removing a molecule from its position in the sorption space to infinity [93].

Dubinin-Radushkevich isotherm model (Equation (11)):

(11) $$\ln q_{e}=\ln Q_{m}-K_{d}\varepsilon^{2}$$

where $q_{e}$ is the amount of SO absorbed, $Q_{m}$ is the theoretical capacity of adsorption (mg g^−1^), $K_{d}$ is a constant related to adsorption energy and ε is Polanyi potential, expressed as Equation (12):

(12) $$\varepsilon =RT\ln\left(1+\frac{1}{C_{e}}\right)$$

where, R, T and $C_{e}$ represent the gas constant (8.314 J mol^−1^ K^−1^), absolute temperature (K) and equilibrium concentration (mg L^−1^), respectively. $K_{d}$ (mol^2^ kJ^−2^) was calculated from the slope of the plot of $\ln q_{e}$ versus $\varepsilon^{2}$ and $q_{m}$ is determined from the intercept. The mean free energy of adsorption E was calculated by using the Equation (13):

(13) $$E=\frac{1}{\sqrt{2K_{d}}}$$

**Figure 10:**
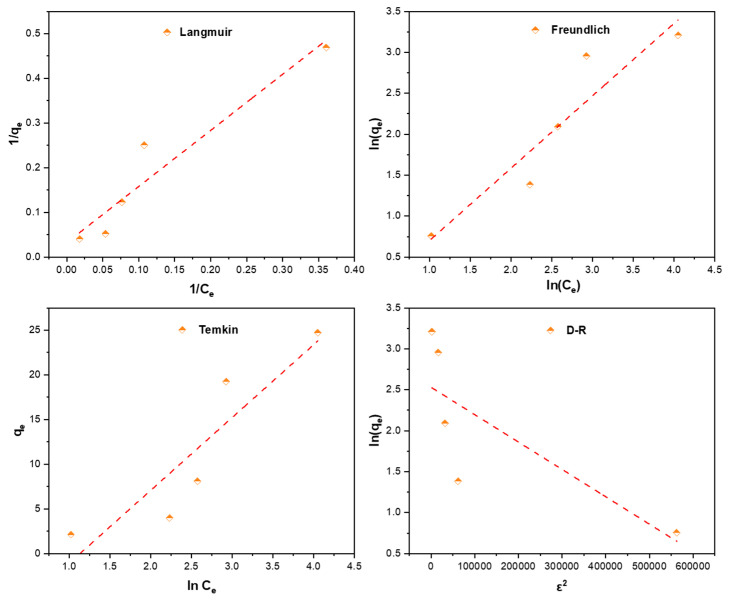
Adsorption isotherms of SO on Alg−PP.

The equilibrium experimental data were fitted using the above reported models and the fitting curves are reported in Figure 10.

Data obtained from fitting process are reported in Table 2. Langmuir equation fits better than the other isotherms with a high R^2^ ≥ 0.924. The empirical data of the Langmuir isotherm also demonstrated a small value of SSE and RMSE which indicates the close fitness of the measured data with the calculated from each model. The maximum adsorption capacity is 30.769 mg/l. The constant b, proving a robust dye-adsorbent bonding, is 0.0258 L/mg. R_L_ value is 0.7 which belonging in the range of 0–1, indicating that SO adsorption onto Alg−PP is a favourable process. The Freundlich isotherm, representative of an adsorption process on heterogeneous surfaces. The value n obtained from the Freundlich model is an indicator of adsorption favourability. A value of n > 1 indicates a favourable nature of adsorption. 1/n gives an idea of the heterogeneity; if 1/n is close to zero a perfect heterogeneous surface is obtained. For our system the value of 1/n is 0.88. Temkin and D-R models didn’t fit the data with good reliability (low R^2^). They deviated from linearity, so they are not reliable in describing adsorption data. Moreover, the value of E was found to be 0.3867 kJ mol^−1^. The value of E is very useful in determining the type of adsorption: if the value is <8 kJ.mol^−1^, then the adsorption is physical in nature, while if it is between 8 kJ mol^−1^ and 16 kJ mol^−1^, then the adsorption is due to ion exchange [94]. In this study, the E value was found to be <8 kJ mol^−1^, so the adsorption possesses a physical nature.

### 2.5. Thermodynamic Study

Free energy change (Equation (14)), enthalpy change (Equation (15)), and entropy change (Equation (16)) for SO adsorption on Alg-PP beads were evaluated using the equation reported hereinafter [95]:

(14) $$\Delta G=-RT\ln K_{d}$$

**Figure 11:**
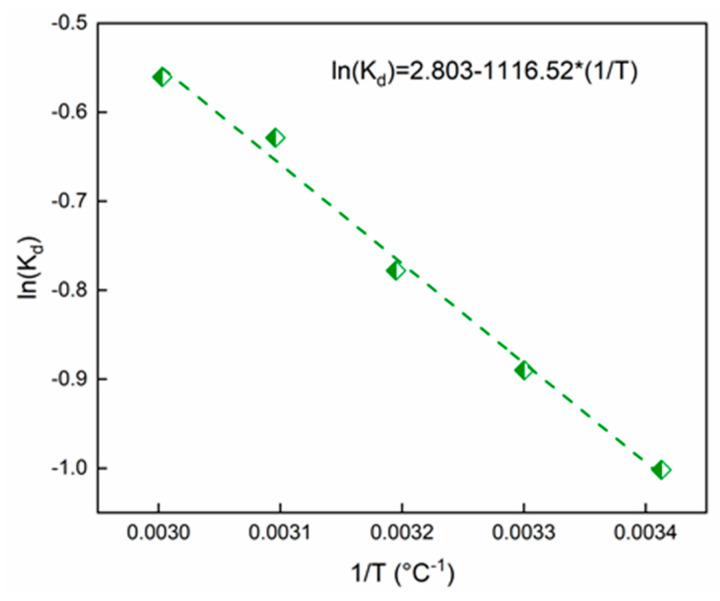
ln($K_{d}$) versus T for the adsorption of SO onto Alg−PP.

(15) ΔG=ΔH−TΔS

(16) $$\ln K_{d}=\frac{\Delta S}{R}-\frac{\Delta H}{RT}$$

where $K_{d}$ is the thermodynamic equilibrium constant at temperature T(K) and $K_{d}$ (L g^−1^) is calculated by using Equation (17):

(17) $$K_{d}=\frac{q_{e}}{C_{e}}$$

where $q_{e}/C_{e}$ is defined as the adsorption affinity [96]. The ln($K_{d}$) was plotted against 1/T (Figure 11):

The thermodynamic values are reported in Table 3.

The values of ΔG < 0 proved the presence of a spontaneous process [82]. The positive enthalpy of the adsorption process at different temperatures indicates that the adsorption of SO on Alg−PP sample is endothermic. So, higher temperature contributed to increase in the activity of the adsorbent and the kinetic energy of the adsorbate, leading to higher removal efficiencies [97]. The ΔS > 0 demonstrates that the process is irreversible with an increasing system disorder and randomness at the solid-liquid interface [98]. Finally, Van der Waals interactions and electrostatic interactions take part in the adsorption process if the enthalpy value lies within 20 kJ/mol [29]. Hydrogen bonding also should be taken into account if values lie inside 25 kJ/mol [99].

### 2.6. Diffusion Coefficients Evaluation

For a spherical system, the total amount of dye adsorbed can be calculated by Equation (18) [100]:

(18) $$\frac{M_{,t}}{M_{,\infty }}=1-\frac{6}{\pi^{2}}\sum_{i=1}^{n}\frac{1}{n^{2}}\exp\left(-\frac{n^{2}\pi^{2}Dt}{R^{2}}\right)$$

where $M_{,t}$ is the amount of dye adsorbed at time t is the equilibrium amount of dye, R is the bead radius while D is the diffusion coefficient. For small times, the equation can be written as Equation (19):

(19) $$\frac{M_{,t}}{M_{,\infty }}=6{\left(\frac{Dt}{R^{2}}\right)}^{0.5}*\left\{\pi^{-0.5}+2\sum_{n=1}^{\infty }ierfc\frac{nR}{\sqrt{Dt}}\right\}-3\frac{Dt}{R^{2}}$$

**Figure 12:**
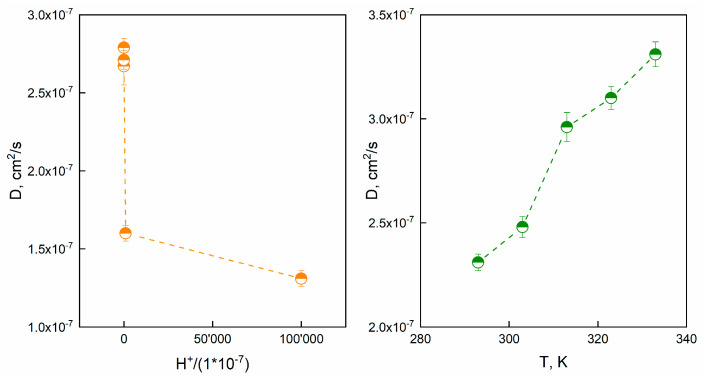
(**a**) D versus normalized [H^+^] and (**b**) D versus T for Alg−PP beads.

The diffusion coefficient D (cm^2^/s) are reported in Table 4 as function of T and pH:

Empirical models were reported to find a correlation between D and either pH or temperature. Figure 12a reports the diffusion coefficient versus H^+^ concentration:

A power-law model was proposed to correlate the diffusion coefficient to the H^+^ concentration (Equation (20)).

(20) $$D=2.8*10^{-7}*{\left(1+\frac{H^{+}}{1*10^{-7}}\right)}^{-0.068}$$

The model forecasts the effect of $H^{+}$ onto diffusion. As far as Figure 12b concerns, diffusion coefficient D (cm^2^/s) as a function of T is represented. Besides, an Arrhenius like equation was used (Equation (21)):

(21) $$D=A*exp(-\frac{E_{a}}{RT})$$

where A is the pre-exponential factor, R is the universal gas constant, $E_{a}$ represents the activation energy, T is the absolute temperature. The activation energy of diffusion ($E_{a}$) was equal to 7.67 kJ/mol. These results suggest that the diffusion coefficient is thermally activated or $H^{+}$ dependent.

### 2.7. Mass Transfer Analysis

**Figure 13:**
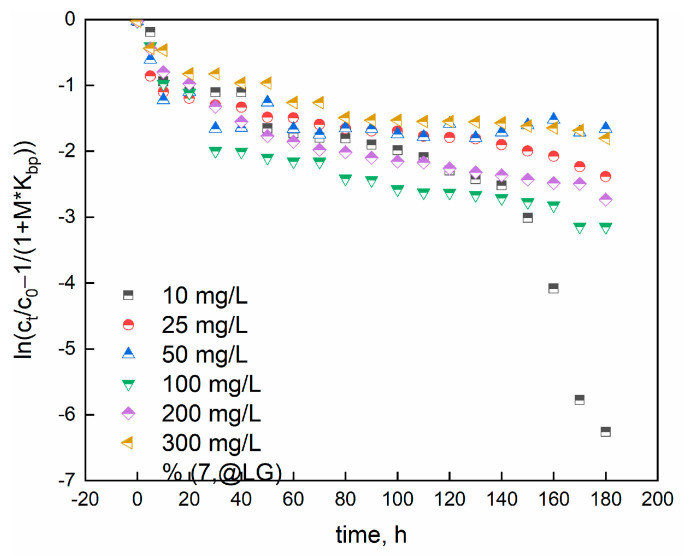
Experimental data fitted through McKay model.

Adsorption data were modelled through the McKay model to investigate whether the adsorption process is controlled by liquid film diffusion (Equation (22)) [101]:

(22) $$\ln\left(\frac{C_{t}}{C_{0}}-\frac{1}{1+MK_{bp}}\right)=\ln\left(\frac{MK_{bp}}{1+MK_{bp}}\right)-\left(\frac{1+MK_{bp}}{MK_{bp}}\right)*\beta *S_{S}*t$$

where $C_{0}$ (mg/L), $C_{t}$ (mg/L), and m (g/L) are the initial dye concentration, the concentration at the time t, and mass/volume ratio, respectively. $K_{bp}$ is McKay constant, obtained by multiplying $q_{max}$ and b (Langmuir constant). The mass transfer coefficient is β and specific surface per unit volume is $S_{S}$. The linearized McKay model allowed to obtain the data reported in Figure 13:

From the plot, β*S_s_ is calculated and shown in Table 5.

The R^2^ coefficients confirm that the McKay model is not appropriate to fully describe the adsorption data. So, the liquid diffusion is not the main rate limiting step.

### 2.8. Bangham’s and Burt model

**Figure 14:**
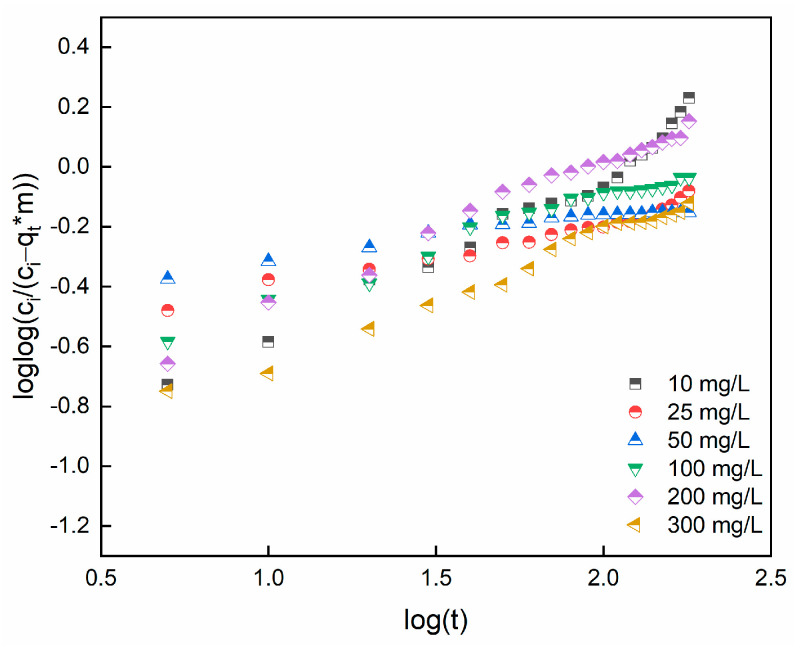
Bangham’s and Burt model data for adsorption of SO.

The Bangham and Burt model can determine if the adsorption process is controlled by pore diffusion [102]. Equation (23) represents the Bangham’s and Burt model in linearized form:

(23) $$loglog\left(\frac{C_{i}}{C_{i}-q_{t}*m}\right)=log\left(\frac{k_{b}*m}{2.303*V}\right)+\alpha_{2}*log(t)$$

where $C_{i}$ (mg/L), m (g), V (L), and $q_{t}$ (mg/g) represent the initial dye concentration (mg/L), mass of adsorbent, volume and adsorption capacity at time t, respectively. $\alpha_{2}$ and $k_{b}$ are constants. Figure 14 reports the linear plots for SO concentrations ranging from 10 to 300 mg/L.

Table 6 reports the model constants and R^2^ coefficient. According to the values, Bangham’s model was not solely a rate-governing stage: it follows that both surface and pore diffusion may control the adsorption process and there is no single rate control step for diffusion into the sorbent pores.

### 2.9. Regeneration Study

**Figure 15:**
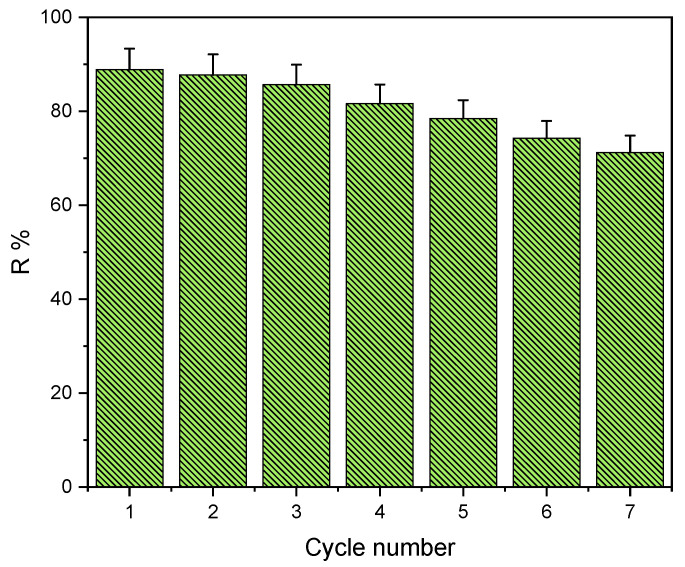
Regeneration studies for Alg−PP beads.

The adsorbent was regenerated to make the process more efficient as well as economical, which significantly increased the process’s economy. When the same adsorbent is reused in multiple adsorption and desorption cycles, stability is generally important. Therefore adsorption/desorption ability was used to determine adsorbent reusability. The results are shown in Figure 15. This was accomplished by carrying out seven consecutive cycles of adsorption/desorption using distilled water at pH = 4 as the adsorbent/eluent. In the first cycle, the desorption efficiency was 88% (from 50 to 6 mg/L), indicating that the adsorbent is suitable for reuse. The removal efficiency decreased from the first cycle to the seventh cycle from 88% to 71% where the variation in concentration was from 50 to 6 mg/L and from 50 to 14.5 mg/L respectively. This finding suggests that Alg−PP beads can be used as a regenerative absorbent.

### 2.10. Adsorption Mechanism

**Figure 16:**
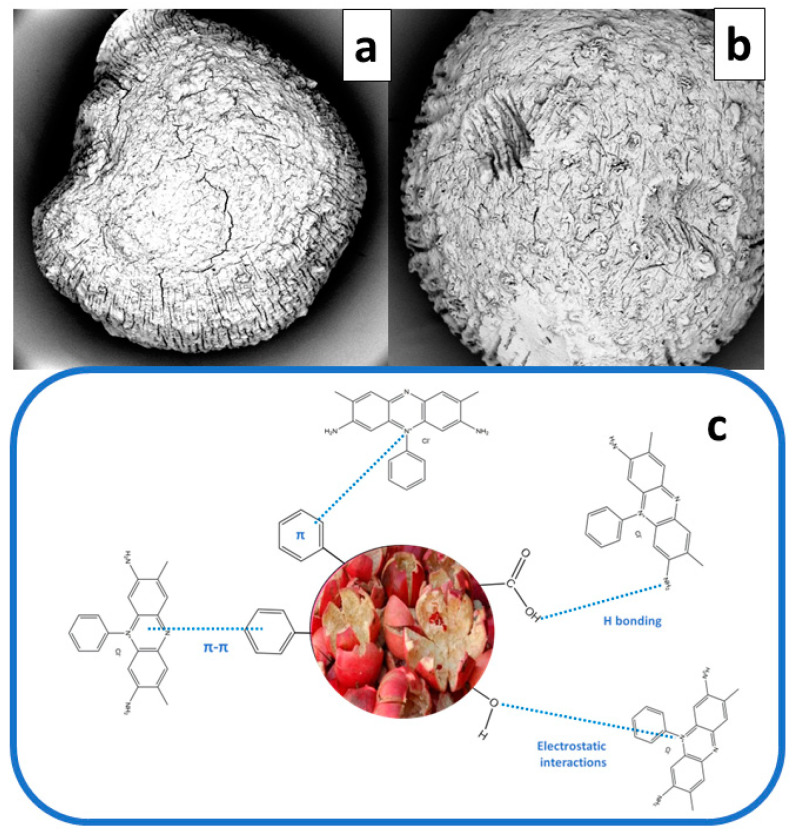
SEM images of Alg−PP before (**a**) and after (**b**) adsorption. SO adsorption mechanisms onto Alg−PP beads (**c**).

The surface structures of the beads before and after dye adsorption are shown in Figure 16. Compared to Alg−PP before adsorption (a), the beads morphology appeared to be irregular and shrivelled with some dumps which could be due to the formation of dye aggregates onto the surface.

The safranin-O dye is a cationic dye able to interacts with lignin, cellulose, hemicellulose, [29]. The free electron pairs of carbonyl oxygen can bind the SO molecule. Electrostatic interactions between cationic SO dye and polar negative groups of adsorbents are supposed to occur. Non-covalent, weak interactions such as van der Waals forces and π-π interaction are established between aromatic rings of safranin O dyes and hemicellulose, lignin, cellulose [103]. The possible physical forces involve: (1) diffusion into pore, (2) H-bonding involving carboxyl and hydroxyl groups bonded directly to a N atom of an −NH_2_ group; (3) π-π interactions; and (4) π^+^-π interaction, bonding between N^+^ of the dye and the aromatic rings of the adsorbent (Figure 16c).

**Figure 17:**
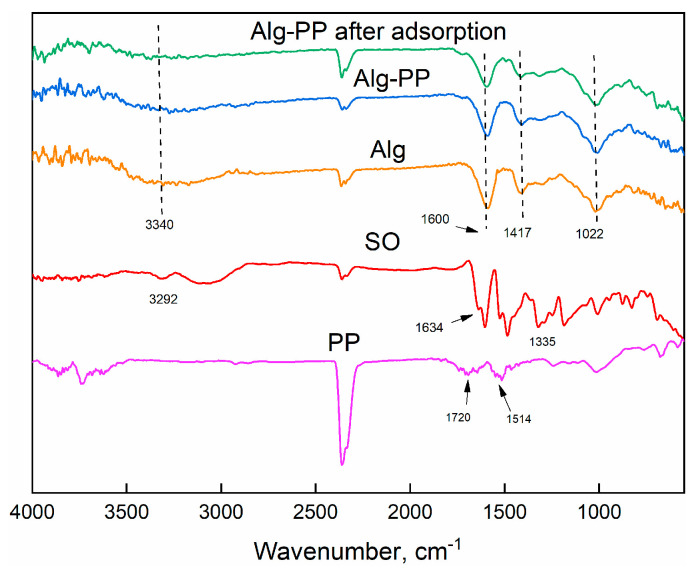
FTIR spectra of PP, SO, Alg, Alg−PP beads before and after the adsorption.

The FTIR spectra shown in Figure 17 are studied for proposing the adsorption mechanisms (Figure 16c). Alg and Alg−PP showed characteristic absorption peaks at around 3340 confirming the stretching shaking of O−H [74]. C−H stretching of CH_2_ small peaks assigned to the peak located at 2911 cm^−1^ and 2850 cm^−1^ are still present. The observed peak at 1710 cm^−1^ in PP spectra corresponds to the C=O stretching of the aldehydes, ketones, and carbonyl groups. The peak 1514 cm^−1^ in PP represents the cellulosic compounds present in the biomaterial [76]. The 1050 cm^−1^ stretching vibration peak of C−O were also observed in all materials. Furthermore, peaks at 1637 cm^−1^ and 1429 cm^−1^ belonged to the antisymmetric and symmetric stretching vibration peak of −COO in sodium alginate [104]. As far as SO spectrum concerns, the bands at 1603 and 1634 cm^−1^ belong to aromatic ring. The peaks at 1335 represented the aromatic-N. However, these newly presented peaks appeared after adsorption of dye. Additionally, the band at 3292 cm^−1^ is attributed to N−H group [105].

The peak centered at 1493 cm^−1^ belongs to the C=C stretching vibrations which could support the presence of an aromatic structure [106]. Also, C–N stretching of aromatic tertiary amine was observed at 1328 cm^−1^ [107]. From the result of FTIR analysis, it was concluded that the Safranin O molecules were biosorbed on the surface of PP by interacting with carboxyl and hydroxyl groups presented.

The adsorption capacity is very promising compared with other adsorbents for SO adsorption as described in Table 7.

## 3. Conclusions

The purpose of this work is to consider the potential of natural materials, the non-modified pomegranate peel (PP) to produce bio-based hydrogels adsorbents. This agro waste material was encapsulated inside sodium alginate hydrogel beads (Alg−PP) through ionotropic gelation to produce a green adsorbent for removing Safranin O from wastewaters. The adsorption of SO cationic dye on Alg−PP beads appeared to be mainly affected by adsorption time, Alg−PP dosage, initial SO concentration and slightly affected by initial pH of SO solution. The dynamic adsorption behavior of SO onto Alg−PP can be well represented by the pseudo-second-order model with an equilibrium adsorption capacity of 26.19 mg/L (C_0_ = 300 mg/L). The Langmuir model showed to greatly fit the adsorption isotherm data proving the favourable nature of the process. The evaluation of thermodynamic parameters proved, since ΔH > 0, the process appeared to be endothermic. The negative value of ΔG showed the feasibility and spontaneity of SO adsorption on Alg−PP beads. The diffusion coefficients were correlated to pH and T. The activation energy of diffusion (E_a_) was equal to 7.67 kJ/mol. The reusability of the Alg−PP beads was examined up to seven cycles. An adsorption mechanism was proposed by considering the chemical and physical phenomena which are supposed to occur. Overall, the hydrogel beads synthesized in this study have effectiveness and can be applied to the removal of cationic dyes in wastewater treatment.

## 4. Materials and Methods

### 4.1. Reagents and Materials

**Figure 18:**
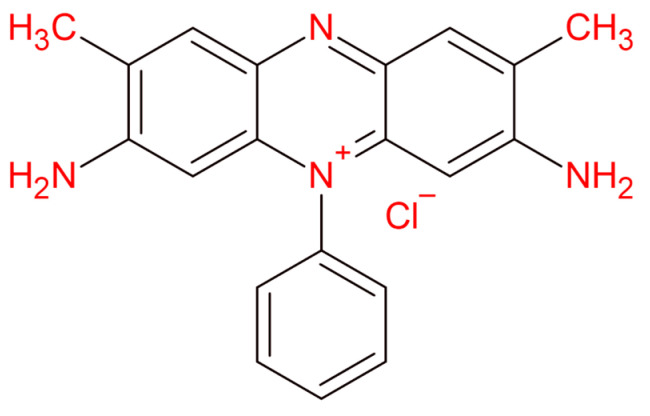
Molecular structure of Safranin O dye.

Sodium alginate (C_6_H_9_NaO_7_, 216.12 g/mol, CAS No. 9005-38-3, >91% purity), hydrochloric acid (HCl, 37%), calcium chloride (CaCl_2_, AR, 96%), sodium chloride (NaCl, AR, 99.5%), sodium hydroxide (NaOH, 99%) were purchased from Sigma Aldrich. Safranin O was purchased from Sigma-Aldrich and was used without further purification. Figure 18 represented the structure of Safranin O dye.

### 4.2. Preparation of Pomegranate Peels Powder

Pomegranate was purchased from a local market in Algeria. The peels of pomegranate were washed with distilled water several times to remove the undesirable residues and dried, crushed and sieved through a 63 μm size before its use without any further processing or chemical treatment.

### 4.3. Preparation of Beads

**Figure 19:**
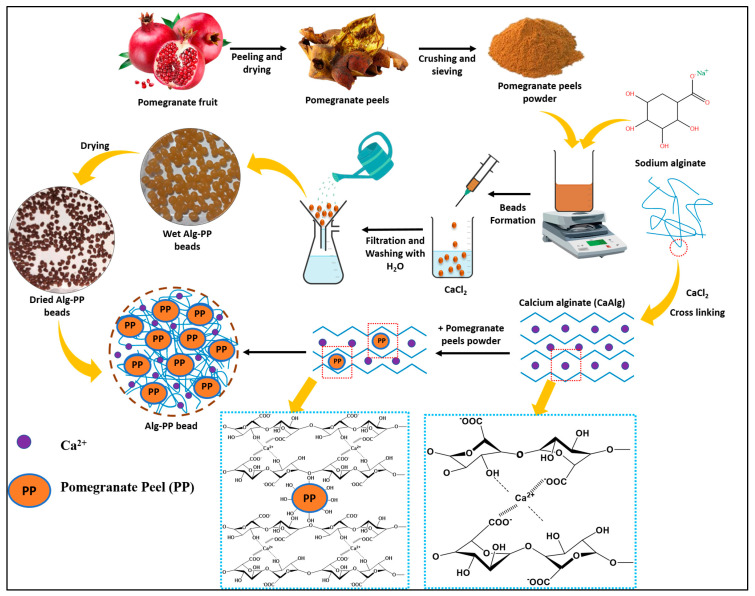
Schematization of Alg−PP preparation.

Sodium alginate water solution (3% *w*/*v*) was prepared by stirring it for 1 h at 50 °C. Then 3 g of pomegranate peels powder was added into the solution until a homogenous Alg−PP dispersion was obtained. The mixture was dropped through a syringe needle into a 4% (*w*/*v*) calcium chloride solution to form beads and left overnight to stabilize. The Alg−PP hydrogel beads were formed through an ion exchange process. The beads were then washed with distilled water to remove the excess of calcium chloride. Finally, they were dried for 24 h in an oven at 50 °C (Figure 19).

### 4.4. Characterization Methods

A gravimetric method was used to evaluate the swelling ability of the hydrogel beads [112]. For 6 h, a certain amount of hydrogel beads was soaked in deionized water at room temperature. The swelling ratio (*S*%) was calculated using the Equation (24) [2]:

(24) $$S\;\left(\%\right)=\frac{W_{w}-W_{d}}{W_{d}}\times 100$$

where $W_{w}$ and $W_{d}$ represent the weights (g) of wet and dried hydrogel beads, respectively.

The water content of the Alg−PP hydrogel was determined by drying beads to a constant weight at 50 °C (Equation (25)):

(25) $$Water\;content\left(\%\right)=\frac{W_{w}-W_{d}}{W_{d}}\times 100$$

where $W_{w}$ and $W_{d}$ are the weights of hydrogel beads before and after drying, respectively.

ImageJ software (Version 1.53t) was used to digitize photographs and determine the mean diameters and distribution of bead sizes of the hydrogel beads. 80 hydrogel beads were measured. Results were reported as mean ± standard deviation.

The surface’s functional groups of the beads were investigated using Fourier transform infrared (FTIR) spectroscopy JASCO FT/IR 4600.

To investigate the surface charge of the hydrogel beads, the pH-drift method was applied to evaluate the point of zero charge (pH_pzc_) [113].

Thermogravimetric analysis (TGA) was carried out in air atmosphere with a Mettler TC-10 apparatus from 30 to 700 °C, at a heating rate of 10 °C/min.

### 4.5. Batch Adsorption Experiments

The dye adsorption experiments were carried out via a batch adsorption process by fixing a stirring rate of 400 rpm. 500 mg of hydrogel beads into 50 mL of different concentrations of dye solutions at room temperature (20 + 2 °C). The adsorbed dye amount onto the hydrogel beads was analyzed by using a UV/VIS spectrophotometer (OPTIZEN POP K LAB Co., Ltd. (Keen Innovative Solutions), Taejon, South Korea) at the wavelength λ= 522 nm. The pH effect on SO adsorption was investigated in the range of 2–10 while the initial dye concentration was fixed at 50 mg/L. The dosage effect of Alg−PP beads was varied from 1 to 40 g/L with a set initial dye concentration of 50 mg/L. The temperature was varied from 30 to 60 °C with a set dye concentration of 50 mg/L. The isothermal adsorption experiments were carried out with 50 mL of dye solutions with different initial concentrations (10–300 mg/L). The adsorption capacity (*q*) and the removal efficiency (*R*%) of SO adsorbed onto hydrogel beads were evaluated through the following Equations (26) and (27):

(26) $$q(\mathrm{mg}/g)=\frac{{(C}_{0}-C)*V}{m}$$

(27) $$R\left(\%\right)=\frac{{(C}_{0}-C_{e})}{C_{0}}*100$$

where $C_{0}(mg/g)$ and $C_{e}(mg/g)$ are the initial and equilibrium concentrations of SO, respectively, m(g) represents the mass of adsorbent, V(L) is the volume.

### 4.6. Reusability Tests

To determine how viable, it would be the reuse of the Alg−PP beads, desorption was carried in batch system. Desorption study was carried by immersing adsorbent Alg−PP beads loaded with SO in 50 mL of distilled water at pH = 4. The mixture was stirred for 24 h. The adsorbent beads were washed several times with distilled water, oven dried at 50 °C and then were reused for adsorption again at room temperature and natural pH. The regenerated adsorbent Alg−PP beads were reused for seven cycles of adsorption-desorption experiments. The percentage of SO removal was calculated by using Equation (27).

### 4.7. Error Analysis

Squares of the errors (*SSE*) and residual root mean square error (*RMSE*) were used to evaluate the fitness of the kinetic and isotherm data with the regression coefficient R^2^, determined from the linearized model. The best fit of the curves is indicated by smaller error analysis values, and vice versa. The equations are expressed as follows:

(28) $$SSE=\sum_{i=1}^{n}({q_{e,exp}-q_{e,cal})}^{2}$$

(29) $$RMSE=\sqrt{\sum_{i=1}^{n}{\left(q_{e,exp}-q_{e,cal}\right)}^{2}}$$

where $q_{e,exp}$ and $q_{e,cal}$ are the equilibrium adsorption capacities (mg/g) measured experimentally and calculated using the isotherm adsorption model, respectively, and n is the number of experimental observations.

## Figures and Tables

**Table 1:** Values of kinetic parameters obtained from fitting process.

Kinetic Model	Coefficients	50 mg/L	100 mg/L	300 mg/L
	$q_{e,exp}$(mg g^−1^)	3.992	8.7957	24.7377
Pseudo-first-order	$q_{e,\;cal}\;$(mg g^−1^)	0.9217	4.6039	12.9416
	$k_{1}\;$(min^−1^)	0.0209	0.0288	0.01813
	$R^{2}$	0.8856	0.6071	0.9387
	SSE	2.5103	23.232	0.9574
	RMSE	0.3961	1.2050	2.2446
Pseudo-second-order	$q_{e,\;cal}\;$(mg g^−1^)	4.0541	9.1166	26.1917
	$k_{2}\;$(g mg^−1^ min^−1^)	0.0595	0.0141	0.0026
	$R^{2}$	0.9997	0.9988	0.9966
	SSE	0.7446	0.7340	0.2573
	RMSE	0.2092	0.2078	0.1230
Elovich	$q_{e,\;cal}$ (mg g^−1^)	0.4074	1.3367	4.3697
	α (mg/g min)	54.8413	7.2104	7.4085
	β (g/mg)	2.4542	0.748	0.2288
	$R^{2}$	0.8304	0.8278	0.9603
	SSE	0.5671	6.2137	13.300
	RMSE	0.1826	0.6045	0.8845
Intraparticle diffusion	$K_{i1}\;$(mg g^−1^ min^−1/2^)	0.5251	1.1135	2.9248
	$C_{1}$ (mg g^−1^)	1.1566	1.2241	2.7464
	$R_{1}^{2}$	0.7865	0.5325	0.8812
	SSE	0.1889	2.7481	2.9092
	RMSE	0.4346	1.6577	1.7056
	$K_{i2}\;$(mg g^−1^ min^−1/2^)	0.1973	1.2676	1.5126
	$C_{2}$ (mg g^−1^)	2.43	0.2905	8.7234
	$R_{2}^{2}$	0.628	0.9580	0.9040
	SSE	0.0153	0.7066	3.7221
	RMSE	0.1236	0.8406	0.8628
	$K_{i3}\;$(mg g^−1^ min^−1/2^)	0.0302	0.1176	0.3266
	$C_{3}$ (mg g^−1^)	3.5733	7.2205	19.8407
	$R_{3}^{2}$	0.9395	0.9580	0.8199
	SSE	0.0041	0.0422	0.5109
	RMSE	0.0177	0.0569	0.2382
	$D_{i}\;$(cm^2^ s^−1^)	1.2.10^−6^	1.2.10^−6^	6.2.10^−7^

**Table 2:** Parameters of isotherm models.

Isotherm Model	Parameters	Value
Langmuir	$q_{m\;}$(mg g^−1^)	30.769
	b(L mg^−1^)	0.0258
	$R_{L}$	0.7725
	$R^{2}$	0.9240
	SSE	0.0096
	RMSE	0.0568
Freundlich	n	1.1312
	$K_{f}\;$((mg g^−1^) * (L mg^−1^)^1/n^)	0.8334
	$R^{2}$	0.8810
	SSE	0.5090
	RMSE	0.4119
Temkin	$a_{T}$ (L g^−1^)	0.3223
	B	8.1594
	$b_{T}\;$(kJ mol^−1^)	298.55
	$R^{2}$	0.8219
	SSE	69.580
	RMSE	4.8159
D-R	$Q_{m}\;$(mg g^−1^)	12.562
	$K_{d}\;$(mol^2^ kJ^−2^)	3.3431
	E(kJ mol^−1^)	0.3867
	$R^{2}$	0.6021
	SSE	1.7020
	RMSE	0.7532

**Table 3:** Thermodynamic parameters for the adsorption of SO onto Alg−PP beads.

Temperature (K)	ΔG (kJ mol^−1^)	ΔH (kJ mol^−1^)	ΔS (kJ mol^−1^ K^−1^)
293	−6.82	9.30	
303	−7.05		
313	−7.28		23.30
323	−7.52		
333	−7.75		

**Table 4:** SO diffusion coefficients as function of pH and T.

Parameters	Value	D, cm^2^/s
pH	2	1.31 ± 0.05 × 10^−7 a^
	4	1.60 ± 0.05 × 10^−7 a^
	6	2.67 ± 0.12 × 10^−7 a^
	8	2.71 ± 0.09 × 10^−7 b^
	10	2.79 ± 0.06 × 10^−7 c^
T	293	2.31 ± 0.04 × 10^−7 a^
	303	2.48 ± 0.05 × 10^−7 b^
	313	2.96 ± 0.07 × 10^−7 b^
	323	3.10 ± 0.055 × 10^−7 c^
	333	3.31 ± 0.06 × 10^−7 c^

For each sample, different superscript letters in the same column indicate that the mean values are significantly different (*p* ≤ 0.05).

**Table 5:** Parameters obtained from McKay’s model.

Concentration, mg/L	β*S_s_, s^−1^	R^2^
10	0.080 ± 0.008 ^a^	0.79
25	0.85 ± 0.10 ^a^	0.81
50	1.03 ± 0.08 ^ab^	0.39
100	0.97 ± 0.09 ^bc^	0.79
200	0.78 ± 0.06 ^c^	0.84
300	0.53 ± 0.03 ^d^	0.83

For each sample, different superscript letters in the same column indicate that the mean values are significantly different (*p* ≤ 0.05).

**Table 6:** Constants of Bangham and Burt’s model.

Concentration, mg/L	α_2_	k_b_	R^2^
10	0.57	0.03	0.97
25	0.22	0.10	0.96
50	0.13	0.17	0.94
100	0.34	0.07	0.97
200	0.48	0.05	0.98
300	0.42	0.04	0.98

**Table 7:** Comparison of maximum dye adsorption capacity between different SO adsorbents.

Adsorbent	Conditions			Q_max_ (mg/g)	References
	pH	T	C_0_ (mg/l)		
Leo−Ca−Alg	8	25	10–30	3.43	[108]
SPION	Natural pH	25	1–15	0.79	[109]
MWCNT/SPION	Natural pH	25	1–15	8.42	
Coal fly ash	9	30	5–50	1.76	[110]
Thuja orientalis	7	20	5–25	0.17	[111]
Alg−PP	Natural pH	25	10–50	30.769	This work

## Data Availability

The data are not publicly available due to ongoing research using a part of the data.
